# Prostanoid Receptor Subtypes and Its Endogenous Ligands with Processing Enzymes within Various Types of Inflammatory Joint Diseases

**DOI:** 10.1155/2020/4301072

**Published:** 2020-11-12

**Authors:** Mohammed A. Al-Madol, Mohammed Shaqura, Thilo John, Rudolf Likar, Reham Said Ebied, Magdi M. Salih, Sascha Treskatsch, Michael Schäfer, Shaaban A. Mousa

**Affiliations:** ^1^Department of Anaesthesiology and Intensive Care Medicine, Charité-University Berlin, Corporate Member of Freie Universität Berlin, Humboldt-Universität zu Berlin, and Berlin Institute of Health, Campus Benjamin Franklin, Berlin, Germany; ^2^Department for Orthopedic and Trauma Surgery, DRK Kliniken Berlin Westend, Berlin, Germany; ^3^Departments of Anaesthesiology and Intensive Care, Hospital Klagenfurt, Klagenfurt, Austria; ^4^Department of Anesthesiology, Theodor Bilharz Research Institute, Giza, Egypt; ^5^Dep. of Histopathology and Cytology, Faculty of Medical laboratory Sciences, Khartoum University, Khartoum, Sudan

## Abstract

A complex inflammatory process mediated by proinflammatory cytokines and prostaglandins commonly occurs in the synovial tissue of patients with joint trauma (JT), osteoarthritis (OA), and rheumatoid arthritis (RA). This study systematically investigated the distinct expression profile of prostaglandin E2 (PGE2), its processing enzymes (COX-2), and microsomal PGES-1 (mPGES-1) as well as the corresponding prostanoid receptor subtypes (EP1-4) in representative samples of synovial tissue from these patients (JT, OA, and RA). Quantitative TaqMan®-PCR and double immunofluorescence confocal microscopy of synovial tissue determined the abundance and exact immune cell types expressing these target molecules. Our results demonstrated that PGE2 and its processing enzymes COX-2 and mPGES-1 were highest in the synovial tissue of RA, followed by the synovial tissue of OA and JT patients. Corresponding prostanoid receptor, subtypes EP3 were highly expressed in the synovium of RA, followed by the synovial tissue of OA and JT patients. These proinflammatory target molecules were distinctly identified in JT patients mostly in synovial granulocytes, in OA patients predominantly in synovial macrophages and fibroblasts, whereas in RA patients mainly in synovial fibroblasts and plasma cells. Our findings show a distinct expression profile of EP receptor subtypes and PGE2 as well as the corresponding processing enzymes in human synovium that modulate the inflammatory process in JT, OA, and RA patients.

## 1. Introduction

Arthritis describes an inflammatory process of a joint which mainly affects the synovial tissue leading to tissue damage and fibrosis [1, 2]. It is very complex and can occur in multiple different ways, e.g., as a consequence of joint trauma (JT), as a leading cause of osteoarthritis (OA), or as a trigger for the autoimmune disease rheumatoid arthritis (RA) [1]. This process is driven by various inflammatory mediators such as tumor necrosis factor *α* (TNF-*α*), interleukin-1*β* (IL-1*β*), and macrophage-colony stimulating factor, as well as prostaglandin E2 (PGE2) and its processing enzyme cyclooxygenase 2 (COX2) [3]. Recently, we demonstrated an overexpression of the synovial proinflammatory mediators such as IL-1*β*, TNF-*α*, and 5-LOX concomitant with the progress of synovial inflammation in JT, OA, and RA diseases [4]. However, the involvement of the different prostanoid receptors EP1-EP4 and their ligands such as prostaglandin E2 (PGE2) as well as their processing enzymes COX2 and mPGES-1 has not been systematically investigated.

Prostaglandin E2 (PGE2) is an arachidonic acid derivative generated by the actions of COX2 (prostaglandin-endoperoxide synthase-2) and microsomal PGES-1 (mPGES-1) and acts as a potent proinflammatory lipid mediator. Emerging evidence suggests that PGE2 and COX-2 are produced during the course of OA and other inflammatory diseases [5]. Furthermore, the induction of both PGE2 and COX-2 was observed in the primary culture of rheumatoid synovial cells after the in vitro treatment with proinflammatory cytokines [5]. The PGE2 binds to a specific group of seven transmembrane domain receptors belonging to the G-protein-coupled “endogenous pyrogen” or EP receptor super family. Activation of these receptors results in physiological and pharmacological effects on cell growth and function [6] as well as in the inflammatory process of arthritis [7]. PGE2 receptors include four subtypes, i.e., the EP1-4 receptors [8, 9]. Largo et al. [10] suggested that endogenous PGE2 might control the propagation of inflammatory processes within the inflamed synovium, whereas Inoue et al. [11] demonstrated in isolated human fibroblasts from OA and RA patients that PGE2 regulated the production of IL-6 and vascular endothelial growth factor via activation of EP2 and EP4 receptors. Moreover, Li et al. [12] showed that stimulation of human articular chondrocytes with PGE2, interleukin-1 (IL-1), and tumor necrosis factor-alpha (TNF-*α*) upregulated the expression of EP2 and EP4 in human chondrocytes in vitro. The authors suggested that these receptor subtypes may initiate the endogenous PGE2-signaling cascade and may serve as an important target for therapeutic regimens in order to prevent the progression of arthritic disease.

Although, there is accumulating data suggesting that PGE2 are implicated in arthritic diseases through the activation of their EP receptors (EP1, EP2, EP3, and EP4), there is no comprehensive study which had thoroughly examined the exact cell types and disease-specific differences in the occurrence of the components of the prostanoid system including EP1-4 receptor subtypes and endogenous ligand PGE2 with its processing enzymes mPGES-1 and COX-2 in various types of synovial disease. We, therefore, systemically investigated the distribution, localization and abundance of PGE-2, its processing enzymes COX-2, and mPGES-1, as well as its corresponding prostanoid receptor subtypes EP1-4 within the synovium of patients with joint trauma (JT), osteoarthritis (OA), and rheumatoid arthritis (RA). Moreover, we determined the number and types of cells expressing these components of the prostanoid system by the use of various cell markers for synovial fibroblasts (P4HB), macrophages/monocytes (CD68), granulocytes (CD15), T lymphocytes (CD3), and plasma cells (Ab-1).

## 2. Materials and Methods

### 2.1. Patients and Synovial Sample Collection

Synovium biopsies of patients were obtained from three different clinics: DRK Clinic Westend Berlin, Landeskrankenhaus Klagenfurt (Austria), and the University Hospital Regensburg. This study was approved by the ethical committees of the Charité-Universitätsmedizin Berlin (Germany), the University Hospital of Regensburg (Germany), and the Landeskrankenhaus Klagenfurt (Austria). Patients' approval was obtained from all three locations, and patients gave their written agreement to the contribution in this study after they were informed about the purpose of this study (EA1/052/12). According to the criteria of the American College of Rheumatology/and European league [13] and the clinical and radiological criteria of OA [14], synovial tissues were collected from patients diagnosed with joint trauma, osteoarthritis, and rheumatoid arthritis. In addition, synovium samples collected from patients who were subjected to a diagnostic arthroscopy were used as a control. During the surgery, synovial tissues were taken from patients and then divided into two pieces, one piece was embedded directly into 4% paraformaldehyde for immunohistochemistry, and the other piece was placed on dry ice for quantitative TaqMan®-PCR for all four groups and then stored at -80°C.

### 2.2. Quantitative TaqMan®-PCR

Total RNA was extracted from synovial tissue samples of 4 patients of each group (*n* = 4) by using the commercially available kit QIAzol Lysis Reagent (Qiagen, Hilden, Germany) as described previously [4]. For cDNA synthesis, 500 ng total RNA was isolated by NanoDrop (Peqlab). Then, 500 ng total RNA was converted to cDNA at 42°C for 1 h using the Omniscript RT Kit (Qiagen, Hilden, Germany) as described previously [4]. The obtained DNA was stored at −20°C. Specific primers for COX-2, mPGEs-1, EP1, EP2, EP3, EP4, and 18s were used (detailed information of primers in Table S1). Finally, quantitative TaqMan®-PCR was performed using a SYBR® Green kit according to the manufacturer's protocol (Applied Biosystems). Amplification was performed with 40 cycles, each consisting of 15 s at 95°C and of 30 s at 60°C. To detect fluorescence specific products for each primer pair, the reaction was carried out at a temperature just below the specific melting temperature (Tm) as described previously [4]. For statistical analysis, experiments were performed in triplicate in order to determine COX-2, EP1, EP2, EP3, EP4, and mPGES-1 mRNA by the delta-delta CT method as described previously [4].

### 2.3. Tissue Preparation and Histological Evaluation

Biopsies of synovial tissue were fixed in 4% (*w*/*v*) paraformaldehyde, dissolved in 0.16 M phosphate buffer solution (PBS) (pH 7.4) for 4 hours, and then cryoprotected overnight at 4°C in PBS containing 10% sucrose. Tissue sections (8 *μ*m) were prepared using a Cryostat (Thermo Fisher, Driesch, Germany) and then mounted onto gelatin-coated slides. Intact synovial tissue was subjected to hematoxylin-eosin histological evaluation identifying the components of lining cell layers and sublining cells as previously described [15, 16]. Cell populations were counted in 3 different tissue sections from 5 patients of each group of diseases by a blinded experimenter (400x magnification) except for the control group (*n* = 4). The lining-layer thickness was assessed by those lining cells only with a visible nucleus in a cross-section at different sites along the entire length of the lining layers and calculated from at least three sections (*n* = 5/group) and three fields per section (400x magnification) [17].

### 2.4. Single and Double Immunofluorescence Staining Procedures

Immunofluorescence staining was performed as described previously [18, 19]. Briefly, the mounted tissue sections were incubated with PBS containing 0.3% Triton X-100, 1% BSA, 10% goat serum, donkey serum, and horse serum (Vector Laboratories, CA, USA) (blocking solution) in order to block nonspecific staining. Then, sections were incubated overnight at 4° C with the following primary antibodies (Table S2): anti-Cox-2, anti-mPGEs-1, anti-PGE2, anti-EP1, anti-EP2, anti-EP3, anti-EP4 alone or in combination with anti-P4HB, anti-CD3, and anti-Ab-1, anti-CD15, or anti-CD68. In addition, some sections were incubated as follows: anti-COX-2/EP3 or anti-PGE2/anti-EP3. Then, the tissue sections were washed in PBS and incubated with Alexa Fluor 594 donkey anti-rabbit antibody alone or in combination with Alexa Fluor 488 donkey anti-goat or with Alexa Fluor 594 donkey anti-mouse (Invitrogen, Germany). Finally, the tissue sections were washed in PBS, and then, the nuclei were stained with DAPI (4′,6-diamidino-2-phenylindole) and mounted in VECTASHIELD (Vector Laboratories). To demonstrate the specificity of staining, the following controls were included: omission of the primary antisera or the secondary antibodies, as described in our previous studies. The images were performed using a confocal laser scanning microscope, LSM510, as described previously [4]. The number of COX-2, PGE2, EP1, EP2, EP3, and EP4 immunoreactive cells was counted by a blinded experimenter in three sections per patient (control *n* = 4 patients, JT *n* = 5 patients, OA *n* = 5 patients, RA *n* = 5 patients).

### 2.5. Statistical Analysis

Data were calculated as means ± SEM. Comparisons between different groups were performed using one-way analysis of variance (ANOVA) followed by Tukey's test in the case of normally distributed data and the Kruskal–Wallis analysis of variance on ranks followed by Dunn's test in the case of data not normally distributed. The data were significantly different if *P* < 0.05. All statistical tests were performed using the Sigma Plot 13.0 statistical software.

## 3. Results

### 3.1. Patients' Characteristics and Synovial Immune Cell Profile

Of 42 patients screened, seven patients had to be excluded from the present study after histological examination because the tissue samples lacked the basic structure of human synovium. The remaining 35 patients were classified dependent on their clinical diagnosis as follows: control (5 patients), JT (9 patients), OA (11 patients), and RA (10 patients). Patients' demographics such as patient's age, gender, disease duration, and medications are presented in Table 1. In addition, our light microscopic analysis of synovial tissues showed that lining-layer thickness, overall cellularity, and vascularity were significantly increased in RA, OA, and JT patients compared to control (*P* < 0.05, one-way ANOVA followed by Tukey's test) (Table 1). Importantly, we further characterized the synovial cellularity in greater detail by double immunofluorescence confocal microscopy for various types of immunocytes and fibroblasts. Indeed, characterization of these cells revealed abundant granulocytes and macrophages within synovium following JT, numerous fibroblasts and macrophages within osteoarthritic synovium, and most prominently plasma cells, fibroblasts, and macrophages within rheumatoid synovium (*P* < 0.05, Kruskal–Wallis ANOVA on ranks followed by Dunn's test) (Figure S1).

### 3.2. Distinct Expression of Prostaglandin E2 (PGE2) as well as Processing Enzymes mPGES-1 and COX-2 in Human Synovium in Various Forms of Inflammatory Joint Disease

**Figure 1:**
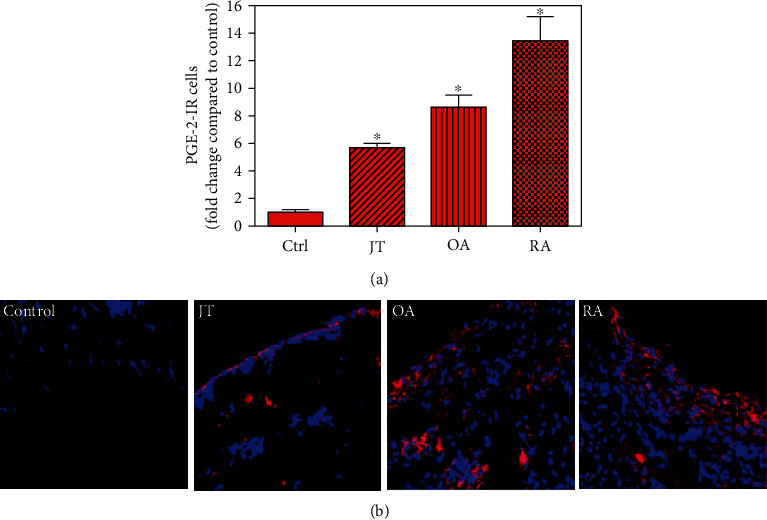
Detection of prostaglandin-E2 (PGE2) in patients with joint trauma (JT), osteoarthritis (OA), and rheumatoid arthritis (RA). (a) Quantitative analysis of immunofluorescence microscopy for synovial PGE2-IR cells relative to the synovium of controls (data are shown as means ± SEM; *P* < 0.05, Kruskal–Wallis ANOVA on ranks followed by Dunn's test). (b) Immunofluorescence microscopy shows that the number of PGE2-IR cells (Texas red fluorescence) is increased in various forms of inflammatory joint disease compared to controls which was most prominent in rheumatoid arthritis. DAPI blue fluorescence shows nuclear staining. Bar = 20*μ*m. (∗*P* < 0.05, compared to control).

**Figure 2:**
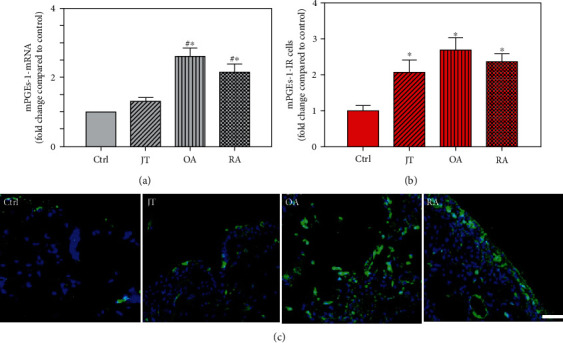
Detection of microsomal prostaglandin E synthase-1 (mPGES-1) mRNA (a) and the number of mPGES-1-IR cells (b) in patients with joint trauma (JT), osteoarthritis (OA) and rheumatoid arthritis (RA). (a, b) Quantification of mPGES-1 mRNA (a) and immunofluorescence positive cells (b) shows that mPGES-1 expression was more prominent in various forms of inflammatory joint disease compared to synovium of controls; however, in patients with RA and OA, it was significantly higher compared to JT (Data are shown as means ± SEM, *P* < 0.05, one-way ANOVA followed by Tukey's test). C: Immunofluorescence microscopy for mPGES (FITC green fluorescence) shows that the number of synovial mPGES-IR cells is increased in various forms of inflammatory joint disease compared to controls which was highest in osteoarthritic synovium. DAPI blue fluorescence shows nuclear staining. Bar = 20*μ*m. (∗*P* < 0.05, compared to control; ^#^*P* < 0.05, compared to JT patients).

**Figure 3:**
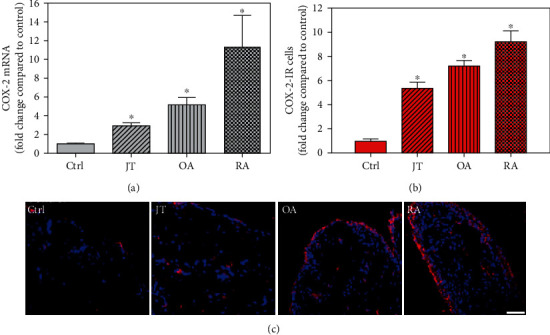
Detection of cyclooxygenase 2 (COX2) mRNA (a) and the number of COX-IR cells (b, c) in patients with joint trauma (JT), osteoarthritis (OA), and rheumatoid arthritis (RA). Quantification of COX-2 mRNA (a) and immunofluorescence positive cells (b) shows that COX-2 expression was more prominent in JT, OR, and RA compared to synovium of controls (data are shown as means ± SEM, *P* < 0.05, one-way ANOVA followed by Tukey's test). (c) Immunofluorescence microscopy for COX-2 (Texas red fluorescence) shows more abundant COX-2-IR cells in JT, OR, and RA compared to control synovium. SEM. DAPI blue fluorescence shows nuclear staining. Bar = 20*μ*m. (∗*P* < 0.05, compared to control).

Quantitative analysis of double immunofluorescence staining of synovial tissues showed that the number of PGE2-IR cells was significantly higher within lining and sublining layers of rheumatoid, osteoarthritic, and joint trauma synovium compared to controls (*P* < 0.05; Kruskal–Wallis ANOVA on ranks followed by Dunn's test); however, it was most abundant in RA patients (Figure 1). Quantitative TaqMan®-PCR investigation showed that mPGES-1 (Figure 2(a)) and COX-2 (Figure 3(a)) specific mRNA was significantly higher in synovial tissue of JT, OA, and RA patients compared to controls (*P* < 0.05; one-way ANOVA followed by Tukey's test); however, it was most prominent in RA synovium. In parallel, mPGES-1- and COX-2-IR cells detected in lining and sublining layers of human synovium were significantly elevated in their number in patients with JT, OA, and RA compared to controls (*P* < 0.05), (Figures 2(b), 2(c), 3(b), and 3(c)). Double immunofluorescence confocal microscopy showed that PGE2 and mPGES-1 as well as COX2 colocalized predominantly in granulocytes and P4HB-IR fibroblasts of patients with JT (data not shown) and in CD68-IR macrophages as well as P4HB-IR fibroblasts of patients with OA and RA (data not shown). All three components of the prostanoid system were separately identified in large populations of Ab-1 plasma cells in RA patients only (data not shown).

### 3.3. Distinct Expression of Prostanoid Receptor Subtypes EP1, EP2, EP3, and EP4 in Human Synovium in Various Forms of Inflammatory Joint Disease

**Figure 4:**
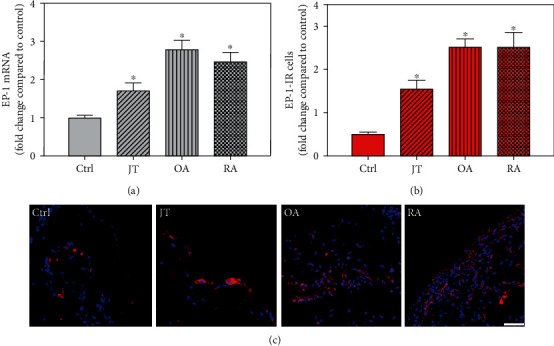
Detection of PGE-2 receptor 1 (EP1 receptor) mRNA (a) and number of EP1-IR cells (b, c) in patients with joint trauma (JT), osteoarthritis (OA), and rheumatoid arthritis (RA). (a) Quantification of EP1 mRNA shows that EP1 mRNA was more prominent in various forms of inflammatory joint disease compared to control synovium (data are shown as means ± SEM, *P* < 0.05, one-way ANOVA followed by Tukey's test). (b) Quantitative analysis of immunofluorescence microscopy for EP1-IR cells relative to control synovium (data are shown as means ± SEM, *P* < 0.05, Kruskal–Wallis ANOVA on ranks followed by Dunn's test). (c) Immunofluorescence microscopy shows more abundant EP1-IR cells (Texas red fluorescence) in JT, OA, and RA compared to control synovium. DAPI blue fluorescence shows nuclear staining. Bar = 20*μ*m. (∗*P* < 0.05, compared to control).

**Figure 5:**
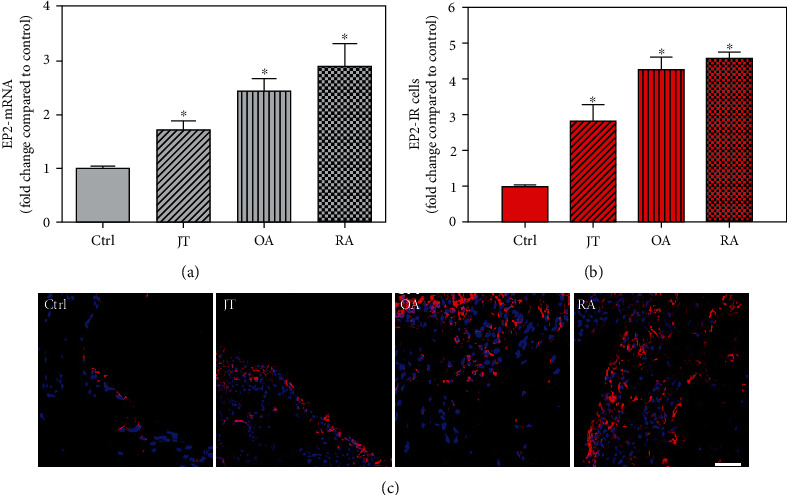
Detection of PGE-2 receptor 2 (EP2 receptor) mRNA (a) and number of EP2-IR cells (b, c) in patients with joint trauma (JT), osteoarthritis (OA), and rheumatoid arthritis (RA). (a) Quantification of EP2 mRNA shows that EP2 mRNA was significantly higher in JT, OA, and RA compared to control synovium (data are shown as means ± SEM, *P* < 0.05, one-way ANOVA followed by Tukey's test). (b) Quantitative analysis of immunofluorescence microscopy for EP2-IR cells relative to the control synovium (data are shown as means ± SEM, *P* < 0.05, Kruskal–Wallis ANOVA on ranks followed by Dunn's test). (c) Immunofluorescence microscopy shows more abundant EP2-IR cells (Texas red fluorescence) in rheumatoid and osteoarthritic synovium compared to JT and control. DAPI blue fluorescence shows nuclear staining. Bar = 20*μ*m. (∗*P* < 0.05, compared to control).

**Figure 6:**
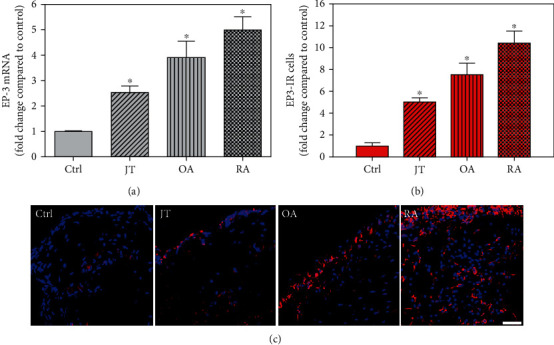
Detection of PGE-2 receptor 3 (EP3 receptor) mRNA (a) and number of EP3-IR cells (b, c) in patients with joint trauma (JT), osteoarthritis (OA), and rheumatoid arthritis (RA). (a) Quantification of EP3 mRNA shows that EP3 mRNA was significantly higher in JT, OA, and RA compared to control synovium (data are shown as means ± SEM, *P* < 0.05, one-way ANOVA followed by Tukey's test) but was more prominent in OA and RA. (b) Quantitative analysis of immunofluorescence microscopy for EP3-IR cells relative to the control synovium (data are shown as means ± SEM, *P* < 0.05, Kruskal–Wallis ANOVA on ranks followed by Dunn's test). (c) Immunofluorescence microscopy shows more abundant EP3-IR cells (Texas red fluorescence) in RA and OA compared to JT and control synovium. DAPI blue fluorescence shows nuclear staining. Bar = 20*μ*m. (∗*P* < 0.05, compared to control).

**Figure 7:**
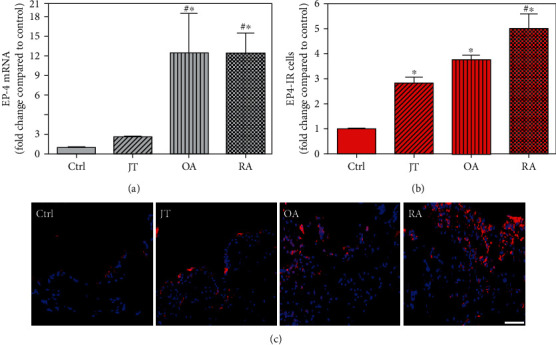
Detection of PGE-2 receptor 4 (EP4 receptor) mRNA (a) and number of EP3-IR cells (b, c) in patients with joint trauma (JT), osteoarthritis (OA), and rheumatoid arthritis (RA). (a) Quantification of EP4 mRNA shows that EP4 mRNA was significantly higher in rheumatoid and osteoarthritic synovium compared to JT and control (data are shown as means ± SEM, *P* < 0.05, one-way ANOVA followed by Tukey's test). (b) Quantitative analysis of immunofluorescence microscopy for EP3-IR cells relative to the control synovium (data are shown as means ± SEM, *P* < 0.05, Kruskal–Wallis ANOVA on ranks followed by Dunn's test). (c) Immunofluorescence microscopy shows more abundant EP3-IR cells (Texas red fluorescence) in JT, OR, and RA compared to control synovium. DAPI blue fluorescence shows nuclear staining. Bar = 20*μ*m. (∗*P* < 0.05, compared to control, ^#^*P* < 0.05, compared to JT).

Quantitative TaqMan®-PCR analysis of specific mRNA for prostanoid receptor subtypes EP1, EP2, and EP3 demonstrated a significant higher expression in the synovium of JT, OA, and RA patients compared to controls (*P* < 0.05) (Figures 4(a), 5(a), and 6(a)), except for the EP4 specific mRNA that was not significantly elevated in JT patients (Figure 7(a)). In parallel, the immunofluorescence confocal microscopy of synovial tissues revealed similar increases in the number of EP1-, EP2-, EP3-, and EP4-IR cells compared to control synovium (Figures 4(b), 4(c), 5(b), 5(c), 6(b), 6(c), 7(b), and 7(c)). Double immunofluorescence confocal microscopy showed that prostanoid receptor subtypes EP2 and EP3 were mainly expressed in CD15-IR granulocytes of JT synovium (e.g., Figure S2) and predominantly identified in CD68-IR macrophages and P4HB-IR fibroblasts of OA (e.g., Figure S3) and RA synovium, whereas they were seen to a large extent in Ab-1 plasma cells only in RA synovium (e.g., Figure S4).

### 3.4. Distinct Expression Profiles of the Prostanoid System in Synovium of JT, OA, and RA Patients

**Figure 8:**
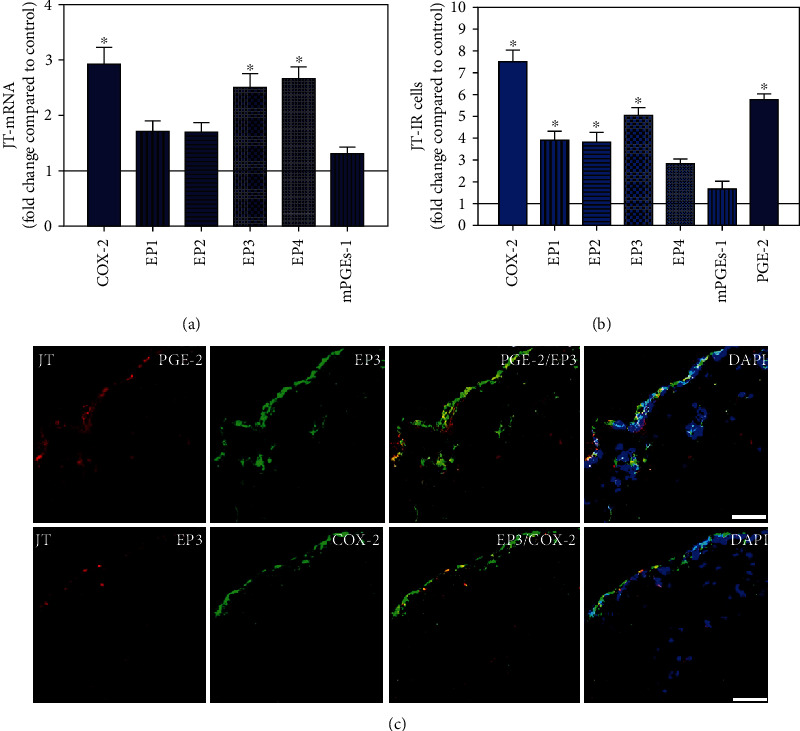
Detection of mRNA (a) and the number of cells immunoreactive for different components of the prostanoid system (PGE2, mPGES, COX-2, and EP1-4) (b) in patients with joint trauma (JT). (a) Quantification of mRNA specific for COX2, EP1-4, and mPGEs-1 in synovium of JT patients. (b) Quantitative comparison of COX2-, EP1-4-, and mPGEs-1-IR cells in synovium of JT patients. Data are shown as means ± SEM. (c) Confocal microscopy of EP3 (Texas red fluorescence; a, b) and mPGES or COX2 (FITC green fluorescence; c, d) double immunofluorescence (e–h) in JT synovium. DAPI blue fluorescence shows nuclear staining. Bar = 40*μ*m.

**Figure 9:**
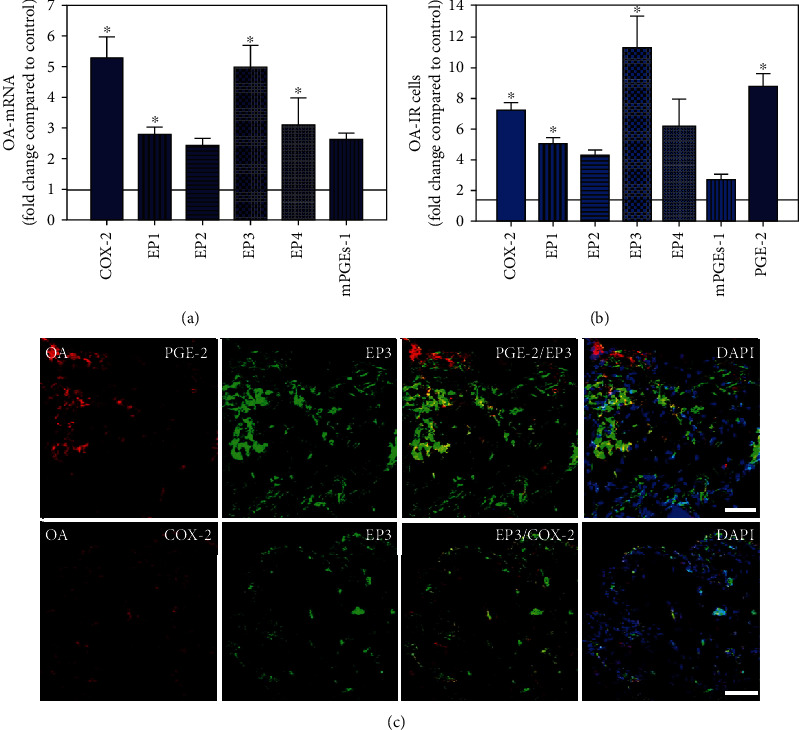
Detection of mRNA (a) and the number of cells immunoreactive for different components of the prostanoid system (PGE2, mPGES, COX-2, and EP1-4) (b) in patients with osteoarthritis (OA). (a) Quantification of mRNA specific for COX2, EP1-4, and mPGEs-1 in synovium of OA patients. (b) Quantitative analysis of COX2-, EP1-4-, and mPGEs-1-IR cells in OA synovium. Data are shown as means ± SEM. (c) Confocal microscopy of EP3 (red fluorescence; a, b) and mPGES or COX2 (green fluorescence; c, d) double immunofluorescence (e–h) in OA synovium. Bar = 40*μ*m. DAPI blue fluorescence shows nuclear staining. Bar = 40*μ*m.

**Figure 10:**
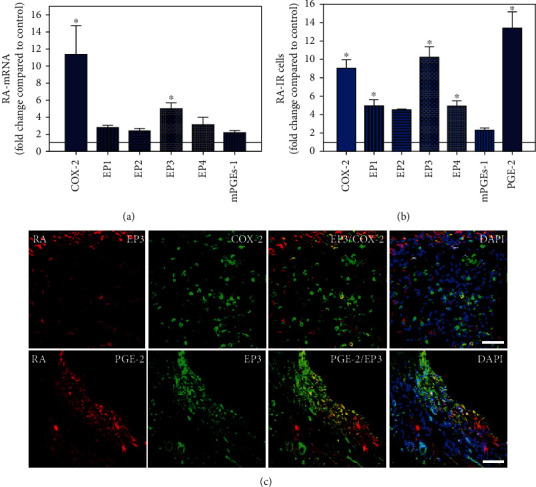
Detection of mRNA (a) and the number cells immunoreactive for different components of the prostanoid system (PGE2, mPGES, COX-2, and EP1-4) (b) in patients with rheumatoid arthritis (RA). (a) Quantification of mRNA specific for COX2, EP1-4, and mPGEs-1 in RA synovium. (b) Quantitative analysis of COX2-, EP1-4-, and mPGEs-1-IR cells in rheumatoid synovium. Data are shown as means ± SEM. (c) Confocal microscopy of EP3 (red fluorescence; a, b) and mPGES or COX2 (green fluorescence; c, d) double immunofluorescence (e–h) in RA synovium. DAPI blue fluorescence shows nuclear staining. Bar = 40*μ*m.

To visualize the expression profiles of the different components of the prostanoid system within a single-patient group (JT, OA, or RA patients), the data for COX-2, EP1-EP4, mPGEs-1, and PGE-2 were converted to and displayed as fold change over control for that individual group (Figures 8–10). Inferring from this, the data show in the synovium of JT patients that the COX-2-expressing cells are the most abundant, followed by EP1-3- and PGE-2-expressing cells (Figure 8). In contrast, in the synovium of OA patients, it emerges that the EP3-expressing cells are the most abundant followed by the PGE-2- and COX-2-expressing cells (Figure 9). Finally, the synovium of the RA patients reveals that the PGE-2-expressing cells are the most abundant, followed by the EP3- and COX-2-expressing cells and, subsequently, by the EP1- and EP4-expressing cells (Figure 10).

## 4. Discussion

Different joint diseases such as joint trauma, osteoarthritis, and rheumatoid arthritis show a distinct inflammatory response within synovial tissue [13, 20]. This study systematically investigated potential differences in the abundance of PGE-2, its processing enzymes COX-2 and mPGES-1, and its corresponding prostanoid receptor subtypes EP1-4 within the synovial tissue of JT, OA, and RA patients in direct comparison to each other and to controls who simply underwent a diagnostic arthroscopy. In line with our mRNA data, immunoreactive cells for PGE2- (13-fold) and its processing enzymes COX-2- (9-fold) and mPGES-1- (2.4-fold) were highest in the synovial tissue of RA patients compared to controls, followed by synovial tissue of OA patients (9-fold, 7-fold, and 2.6-fold, respectively) and of JT patients (5.8-fold, 7.5-fold, and 2-fold, respectively). Identification of the corresponding prostanoid receptor subtypes within the synovial tissue of these patients revealed that the EP3 prostanoid receptor subtype was the most abundant in all three groups of patients (RA patients: 10-fold; OA patients: 11-fold; and JT patients: 5-fold) followed by the other prostanoid receptor subtypes (EP1-EP3) which differed not much among each other (RA: ca. 5-fold; OA: ca. 4-fold; and JT: ca. 3-fold). Further identification of the most abundant immune cells within synovial tissue expressing the different components of the prostanoid system showed predominantly granulocytes (CD15) and fibroblast-like synoviocytes (P4HB) in JT patients, mainly macrophages (CD68) and fibroblast-like cells (P4HB) in OA patients, and mostly plasma (AB-1), macrophages (CD68), and fibroblast-like-synoviocytes (P4HB) in RA patients. Taken together, direct comparison of the different components of the prostanoid system within the synovial tissue of JT, OA, and RA patients in a single experimental approach showed the highest abundance in RA followed by OA and JT patients, a predominant expression of the EP3 receptor in all three groups and a distinct profile of immune cells involved.

For a long time, it has been known that prostaglandins such as PGE-2—assessed by a radioimmunoassay—are elevated in the synovial fluid of patients suffering from RA, OA, or JT [21] and that mononuclear cells [22], obtained from the synovial fluid of RA patients, as well fibroblast-like synoviocytes, isolated from the synovium of RA [23–26] and OA patients [27], release PGE-2. Further experiments demonstrated that inhibition of the COX enzyme prevented the production and release of PGE-2 in rheumatoid synovial fibroblasts [28], and the PGE-2 processing enzymes were identified by RT-PCR or Western blot as COX-1, COX-2, and PGES-1 within the synovium of RA [23, 25, 28, 29] and of OA patients [12, 30, 31]. Only recently, COX-1, COX-2, and PGES-1 were demonstrated by immunohistochemistry within the synovium of RA patients [32, 33]. Our findings go beyond these studies in that we systematically compared among JT, OA, and RA patients the synovial expression (mRNA) and abundance (immune fluorescence microscopy) of PGE-2 with its processing enzymes COX-1, COX-2, and PGES-1 within a single experimental approach. These components of the prostanoid system were identified in all three groups of patients; however, they were highest in RA patients, followed by OA and JT patients.

In light of these findings, we further assessed which prostanoid receptor subtypes were expressed most prominently within the synovium of JT, OA, and RA patients. Intriguingly, the EP3 prostanoid receptor subtype was the most abundant in all three groups of patients (5- to 11-fold over control), followed by a similar, but lower expression of the EP1, EP2, and EP4 receptors. Consistent with previous studies in mainly rheumatoid synovial fibroblasts [34, 35], we found an enhanced expression (3- to 5-fold) of the EP1, EP2, and EP4 receptors within the synovium of JT, OA, and RA patients. Experimental studies with knockout mice indicated that the EP4 receptor knockout was able to significantly decrease the incidence and severity of arthritis, whereas the EP1-EP3 receptors essentially do not contribute to the inflammatory process of arthritis [36, 37]. What stands out from our results is that the EP3 receptor was highly prominent in its abundance within the synovial tissue of all three groups of patients. Previous experimental studies with EP3 receptor knockout mice demonstrated a significant antinociceptive effect in which the putative mechanism is still unclear [2, 38]. More recently, EP3 receptors have been shown in fibroblasts and peripheral nerve bundles of synovial tissue in OA patients, and their activation led to profound antinociceptive effects in an experimental study, in contrast to the described proinflammatory effects of EP2 and EP4 receptors [39]. Also, Attu et al. [40] stated that prostaglandin E2 exerts catabolic effects in osteoarthritis cartilage via the EP4 receptor. Previous studies reported that the PGE2 signal through the EP2 receptor promotes the growth of articular chondrocytes [41] or enhances the regeneration of injured articular cartilage [42].

Our immunofluorescence confocal microscopy analysis revealed that prostanoid receptor subtypes (EP1-4) were expressed predominantly in synovial granulocytes (CD15) of JT patients, in fibroblast-like synoviocytes (P4HB) and macrophages (CD68) of OA patients and in macrophages as well as plasma cells of RA patients. This is consistent with a previous study by Dechanet et al. [43] which stated that plasma cells were identified as clusters in close contact with macrophages, synoviocytes, and CD8^+^ T-cells within rheumatoid synovium [43]. Using double immunofluorescence confocal microscopy, we identified EP1 and EP4 also in high populations of mature B-lymphocytes such as plasma cells within synovium of RA patients extending previous studies by Fedyk et al. [44] which demonstrated mRNA encoding EP1, EP3, and EP4 receptors in normal and transformed B-lymphocytes. Collectively, the present findings suggest that prostanoid receptor subtypes and PGE2 with its processing enzymes mPGES-1 and COX-2 expressed in distinct cell populations may differentially mediate the inflammatory process within JT, OA, and RA synovium.

Our study certainly has its limitations in that the number of patients' samples is low and does not necessarily allow generalization of our results to a larger population of patients. Moreover, the inflammatory process during JT, OA, and RA arthritis is a dynamic process, and our synovial tissue samples obtained give only a cross-sectional picture of these patients at a specific moment of their disease. In addition, differences in the patients' daily medications might have influenced the results. Thus, one can only cautiously draw conclusions from our results. An advantage of our study though is that we compared the different synovial tissues within a single experimental approach so that experimental conditions were kept similar among the different groups.

Taken together, our direct comparison of the different components of the prostanoid system within the synovial tissue of JT, OA, and RA patients in a single experimental approach showed the highest abundance in RA followed by OA and JT patients, a predominant expression of the EP3 receptor in all three groups and a distinct profile of immune cells involved.

## Figures and Tables

**Table 1:** The clinical and histological data characterizing the patients with joint trauma, osteoarthritis, and rheumatoid arthritis.

Patient Nr.	Control (*n* = 5)	JT (*n* = 9)	OA (*n* = 11)	RA (*n* = 10)
Age (years)	65 (±16,8)	48 (±17)	72 (±6)	64.9 (±17)
Sex (F/M)	1/4	6/4	7/3	4/6
Disease duration				
≤1 year	5/5	7/10	3/10	—
>1 year	—	3/10	7/10	10/10
Drugs: NSAIDs (Diclofenac, Ibuprofen, Profenid, Paracetamol, and Dipyrone)	5/5	8/10	9/10	8/10
Etoricoxib	NA	1/9	4/10	1/10
Prednisolon (dexamethasone)	NA	NA	NA	8/10
Lining-layer thickness (cell layers)	1 (1 : 2)	2 (1: 3)	3∗ (3: 4)	4∗ (3: 5)
Overall cellularity (cells/mm^2^)	45 (44; 49)	119∗ (97; 138)	275∗^#^ (220; 285)	390∗^#^ (358; 474)
Vascularity (vessels)	2 (1; 3)	3 (3; 5)	6∗ (5; 7)	6∗ (3; 8)

∗*P* < 0.05, compared to the control, ^#^*P* < 0.05 compared to JT. One-way ANOVA followed by Tukey's test.∗*P* < 0.05, compared to control; ^#^*P* < 0.05, compared to JT.

## Data Availability

Shaaban Mousa as a corresponding author can make data available on request through the authors themselves.

## Supplementary Materials

Supplementary Materials Table S1: characterization of the primers used for PCR. Table S2: characterization of primary antibodies used. Figure S1: quantitative analysis of immunofluorescence microscopy of various types of immune cells and fibroblasts showing the number of overall synovial cellularity. Note that the number of immune cells and fibroblasts within OA and RA synovium was significantly higher compared to JT patients and controls (*P* < 0.05, Kruskal–Wallis ANOVA on ranks followed by Dunn's test). Moreover, granulocytes and macrophages were most prominent in JT, but fibroblasts and macrophages were more abundant in OA. In RA patients, the plasma cells, fibroblasts, and macrophages were the most abundant compared to controls (*P* < 0.05, Kruskal–Wallis ANOVA on ranks followed by Dunn's test). Data are shown as means ± SEM. (∗*P* < 0.05, compared to control, ^#^*P* < 0.05, compared to JT, and *P* < 0.05, compared to OA), (in Granulocyte; ^§^*P* < 0.05, compared to OA and RA). Figure S2: double immunofluorescence confocal microscopy of prostaglandin E2 receptor 3 (EP3) (red fluorescence; a, e, and i) with CD68 (b), P4HB (f), or CD15 (j) (green fluorescence) in synovial tissue of patients with joint trauma (JT). Note that the majority of EP3 immunoreactive cells coexpress CD15 (granulocyte) and P4HB (fibroblast) but not CD68 (macrophage) in JT synovium. Bar = 20*μ*m. Figure S3: double immunofluorescence confocal microscopy of PGE-2 receptor 3 (EP3) (red fluorescence; a, e, and i) with CD68 (b) or P4HB (f) (green fluorescence) in synovial tissue from patients with osteoarthritis (OA). Note that a high population of EP3 immunoreactive cells coexpress P4HB or CD68 in OA synovium. Bar = 20*μ*m. Figure S4: double immunofluorescence confocal microscopy of PGE-2 receptor 3 (EP3) (red fluorescence; a, e, and i) with CD68 (b), P4HB, or Ab-1 (plasma cells) (f) (green fluorescence) in synovial tissue from patients with rheumatoid arthritis (RA). Note that a high population of EP3 immunoreactive cells coexpress CD68 (macrophage), P4HB (fibroblast), or Ab-1 (plasma cells) in OA synovium (a–d). Bar = 20*μ*m.
