# Supplementary material for: Prostanoid Receptor Subtypes and Its Endogenous Ligands with Processing Enzymes within Various Types of Inflammatory Joint Diseases
Source: Mediators Inflamm. 2020 Nov 12;2020:4301072. doi: 10.1155/2020/4301072 (PMC7676943; doi:10.1155/2020/4301072)

Supplemental table 1

List of Primers for Quantitative Taqman RT-PCR

Gene	Access. Nr.	Forward	Reverse
COX-2	NM_000963.3	GCTCAAACATGATGTTTGCA TTC	TCATAAGCGAGGGCCAGC
mPGE s-1	NM_004878	CCTGGGCTTCGTCTACTCCTT T	CAGGTAGGCCACGGTGTGT
EP1	NM_ 000955.2	CTTCGGCCTCCACCTTCTTT	GCCACCAACACCAGCATTG
EP2	NM_000956.3	TATCATGACCATCACCTTCGC CGT	CCTAAGAGCTTGGAGGTCCC ATT
EP3	NM_00112604 4.1	GTGCTGTCGGTCTGCTG	CTTTCTGCTTCTCCGTGTG
EP4	NM_000958.2	CATCTGCTCCATCCCGCT	GGATGGCCTGCAAATCTGG
S18	NR_046237	AAACGGCTACCACATCCAAG	CCTCCAATGGATCCTCGTTA

Supplemental table 2

Characterization of primary antibodies used.

Antigen	Manufacturer, Species, Type, Catalogue Number	DILUTION
COX-2	Abcam, Cambridge, UK, Polyclonal Rabbit IgG Anti-human # ab6665	1:300
COX-2	Cayman Chemical, MI, USA, Monoclonal mouse IgG against human # Clone CX229.	1.100
PGE2	Cayman Chemical, MI, USA, Monoclonal mouse IgG against human # Item No. 10009814.	1:200
mPGEs-1	Cayman Chemical, MI, USA, Monoclonal mouse IgG against human # Item No. 10004350.	1:200
EP1-EP4	Gift from DR. R Nüsing, Institute of Clinical Pharmacology, Johann Wolfgang Goethe.University, Frankfurt, Germany, Polyclonal Rabbit IgG Anti-human	1:300
CD15	Dako; Glostrup. Denmark Monoclonal Mouse IgM against human # Clone C3D-1:Nr. M0733	1:200
CD68	Dako; Glostrup. Denmark Monoclonal Mouse IgG against human # M 0814 clone KP1(macrophages)	1:200
Prolyl-4-Hydroxylase beta	Acris Antibodies, Inc., San Diego, CA, USA Monoclonal Mouse IgG_1 against human # Clone 3-2B12	1:400
Plasma cell Ab-1	Thermo Scientific , CA, USA Monoclonal Mouse IgG_2a against human # Clone LIV3G11	1:500
CD3	Dako; Glostrup. Denmark Monoclonal Mouse IgG against human # M 7193 Clone PC3/188A (T Cell)	1:500

Supplemental figure 1

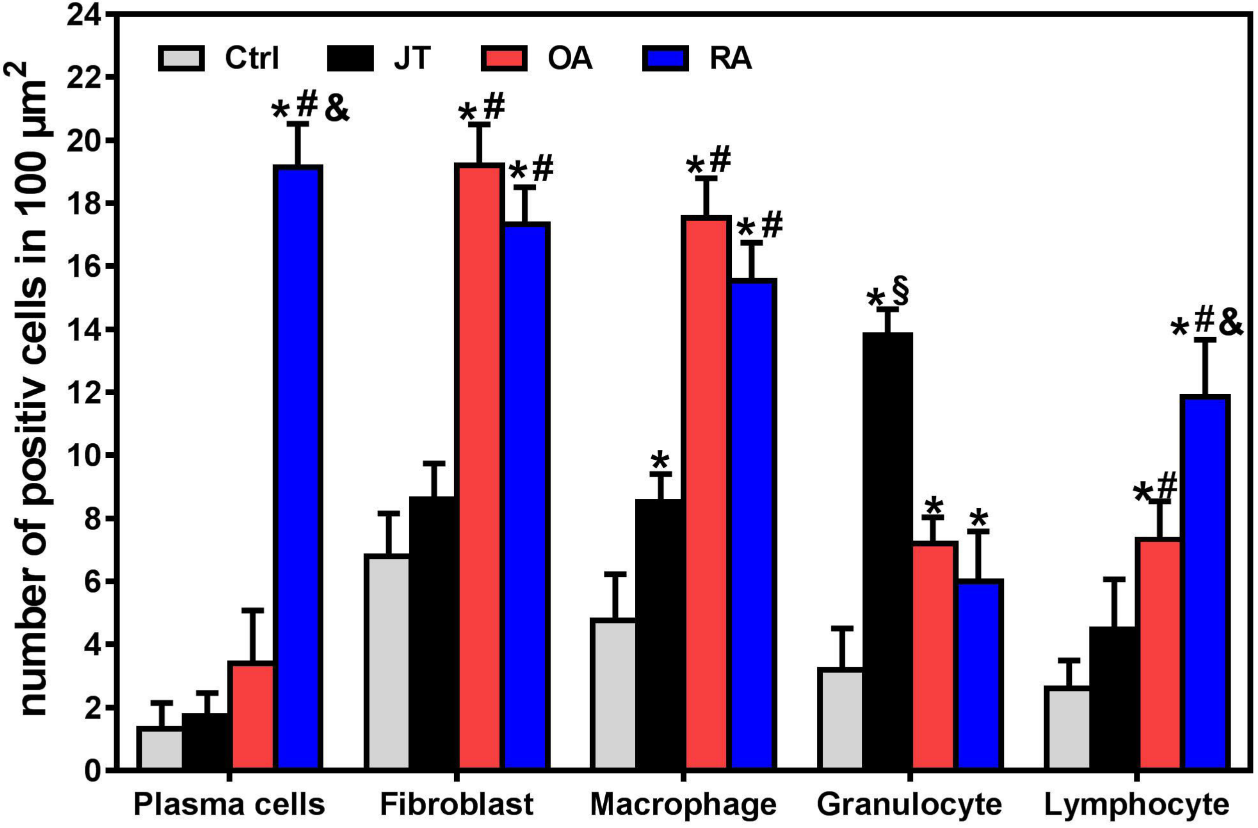

Supplemental figure 2

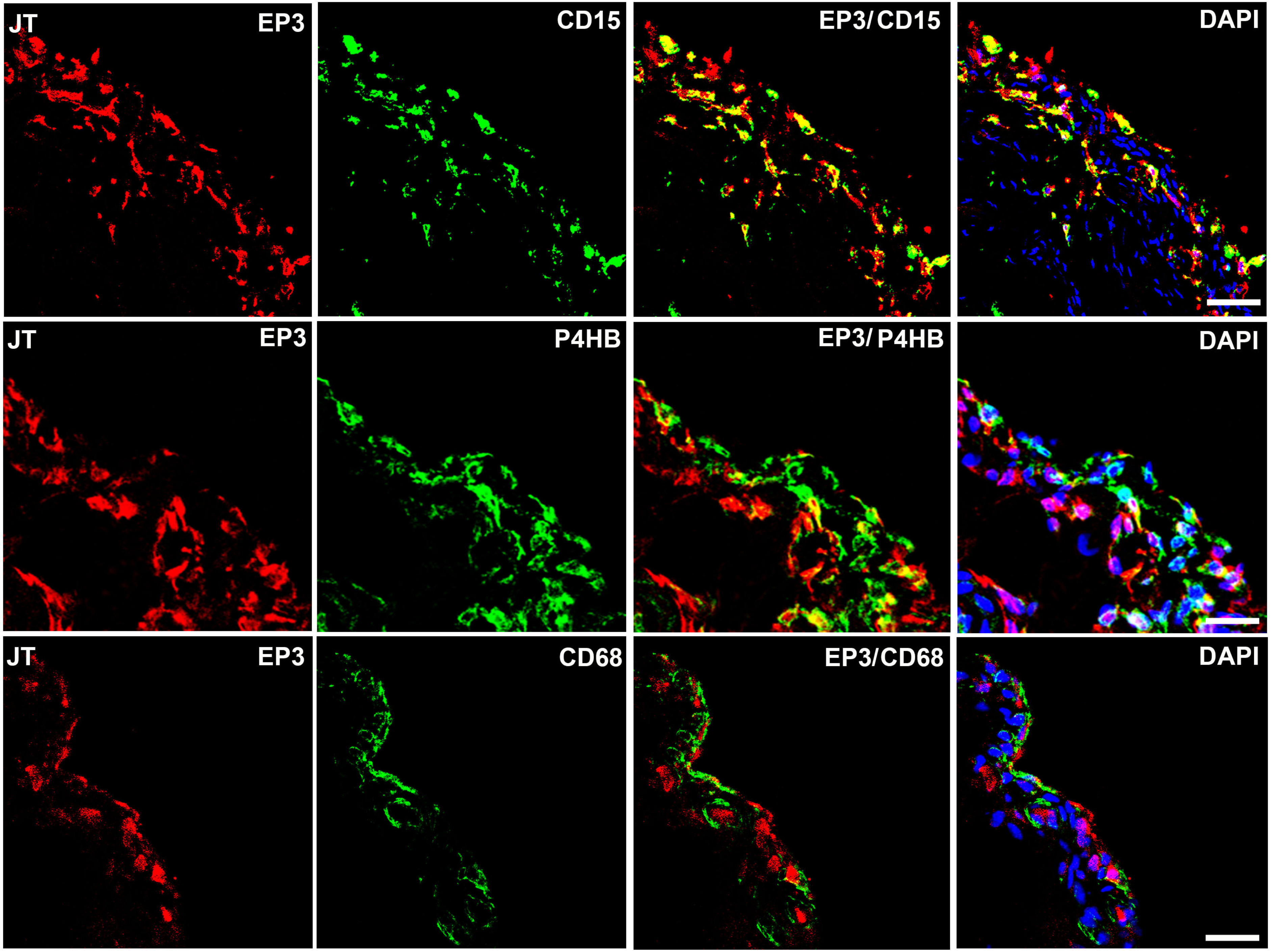

Supplemental figure 3

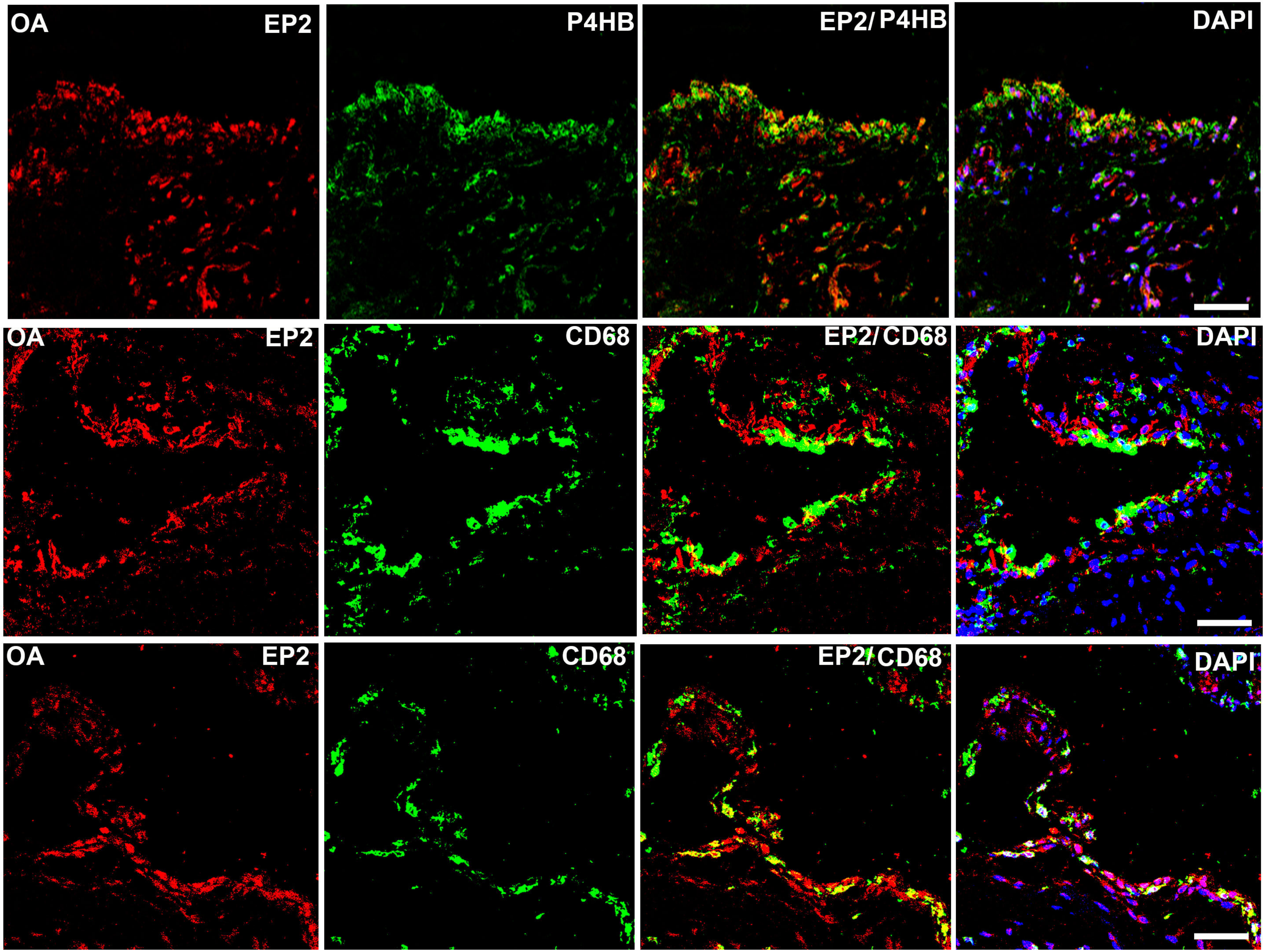

Supplemental figure 4

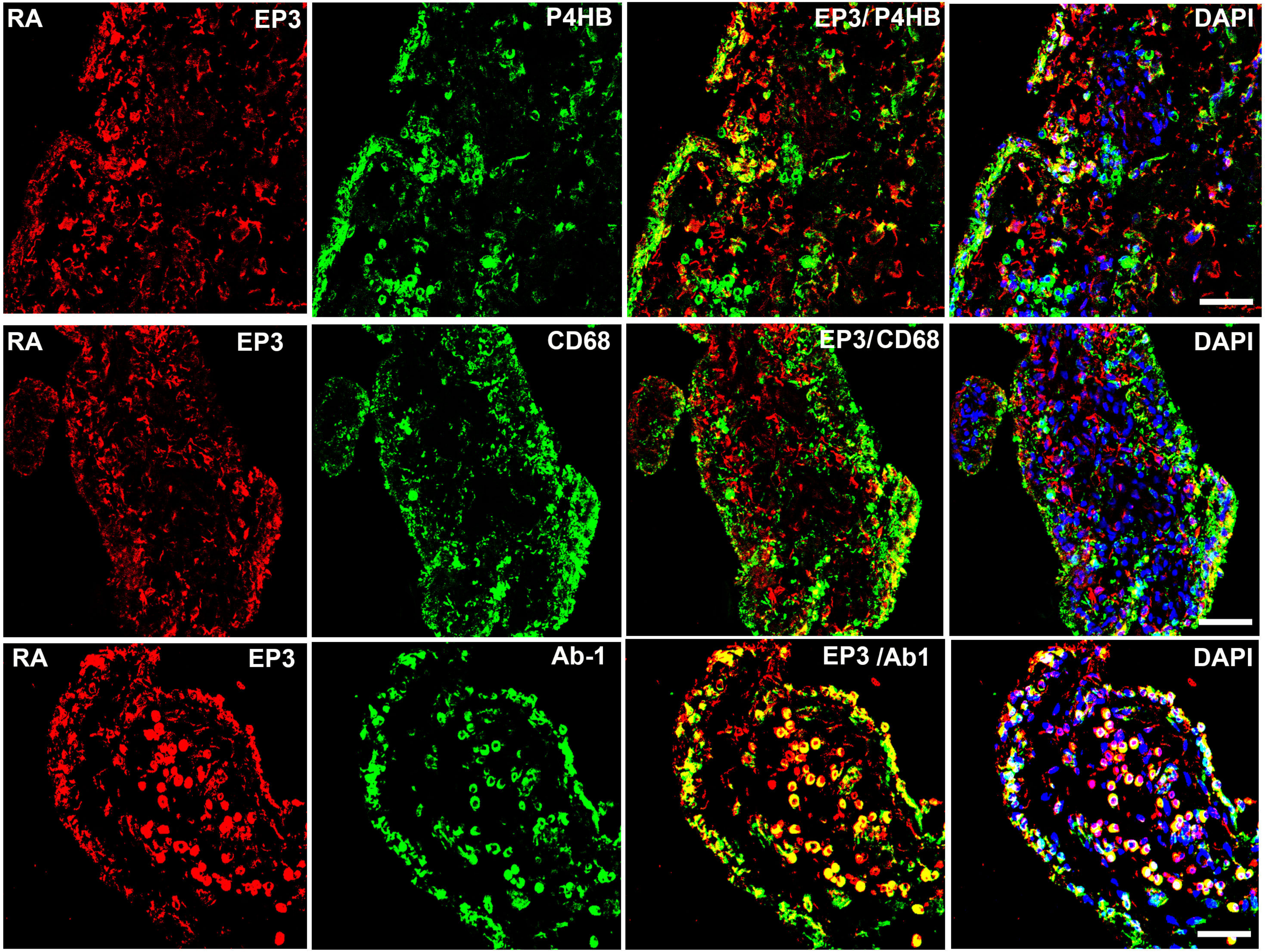

Supplement: Supplementary Materials — Table S1: characterization of the primers used for PCR. Table S2: characterization of primary antibodies used. Figure S1: quantitative analysis of immunofluorescence microscopy of various types of immune cells and fibroblasts showing the number of overall synovial cellularity. Note that the number of immune cells and fibroblasts within OA and RA synovium was significantly higher compared to JT patients and controls (P < 0.05, Kruskal–Wallis ANOVA on ranks followed by Dunn's test). Moreover, granulocytes and macrophages were most prominent in JT, but fibroblasts and macrophages were more abundant in OA. In RA patients, the plasma cells, fibroblasts, and macrophages were the most abundant compared to controls (P < 0.05, Kruskal–Wallis ANOVA on ranks followed by Dunn's test). Data are shown as means ± SEM. (∗P < 0.05, compared to control, #P < 0.05, compared to JT, and P < 0.05, compared to OA), (in Granulocyte; §P < 0.05, compared to OA and RA). Figure S2: double immunofluorescence confocal microscopy of prostaglandin E2 receptor 3 (EP3) (red fluorescence; a, e, and i) with CD68 (b), P4HB (f), or CD15 (j) (green fluorescence) in synovial tissue of patients with joint trauma (JT). Note that the majority of EP3 immunoreactive cells coexpress CD15 (granulocyte) and P4HB (fibroblast) but not CD68 (macrophage) in JT synovium. Bar = 20μm. Figure S3: double immunofluorescence confocal microscopy of PGE-2 receptor 3 (EP3) (red fluorescence; a, e, and i) with CD68 (b) or P4HB (f) (green fluorescence) in synovial tissue from patients with osteoarthritis (OA). Note that a high population of EP3 immunoreactive cells coexpress P4HB or CD68 in OA synovium. Bar = 20μm. Figure S4: double immunofluorescence confocal microscopy of PGE-2 receptor 3 (EP3) (red fluorescence; a, e, and i) with CD68 (b), P4HB, or Ab-1 (plasma cells) (f) (green fluorescence) in synovial tissue from patients with rheumatoid arthritis (RA). Note that a high population of EP3 immunoreactive cells coexpress CD68 [file 4301072.f1.pdf]
